# Supplementary material for: Identification of Phytaspase Interactors via the Proximity-Dependent Biotin-Based Identification Approach
Source: Int J Mol Sci. 2021 Dec 4;22(23):13123. doi: 10.3390/ijms222313123 (PMC8658550; doi:10.3390/ijms222313123)
Supplement: Supplementary file 1 [file ijms-22-13123-s001.zip › ijms-1472844-supplementary.pdf]

Figure S1. Lack of significant biotinylation signals in the apoplastic fractions from NtPhyt-Turbo- and SP-Turbo- N. benthamiana leaves.

Western blot analysis with streptavidin-HRP detection of apoplastic (Ap) and intracellular fractions (ICF) obtained from leaves producing either NtPhyt-Turbo (Phyt) or SP-Turbo (SP) proteins. Positions of molecular weight protein markers are indicated on the left.

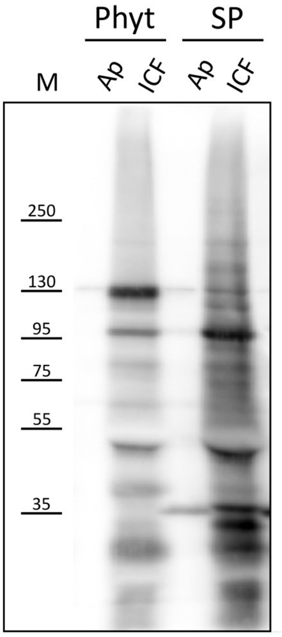

Figure S2. Sequence coverage of the proteins listed in Table 1 with mass spectrometry-identified peptides.

1) endoplasmin homolog XP_019239585.1

Match to: **NbE44072412.1** Score: **134** Expect: **6e-009**
NbE44072412.1 endoplasmin homolog (XP_019239585.1)

Nominal mass (M_r): **87552**; Calculated pI value: **4.92**
NCBI BLAST search of NbE44072412.1 against nr
Unformatted sequence string for pasting into other applications

Variable modifications: Carbamidomethyl (C),Oxidation (M)
Cleavage by Trypsin: cuts C-term side of KR unless next residue is P
Number of mass values searched: **78**
Number of mass values matched: **22**
Sequence Coverage: **32%**

Matched peptides shown in **Bold Red**

1 MRKWTIPSVL FLLCLLFLLP DQGRRIQANA EAESDVPVDP PKVEEKFGAI
51 PHGLSTDSDV VKRESESMSR KTLRADAEK**F EFQAEVSR**LM DIIINSLYSN
101 K**DIFLR**ELIS NASDALDKIR **FLSLTDKEVL GEGDNTK**LEI QIKLDKEKKI
151 LSIRDRGIGM TKEDLIKNLG TIAKSGTSAF VEKMQTSGDL NLIGQFGVGF
201 YSVYLVADYV EVISKHNDDK **QYVWESKADG AFAISEDVWN EPLGR**GTEIR
251 LHIR**DEAGEY LDEYK**LKDLV KKYSEFINFP IYLWASKEVE KEVPADEDES
301 SDEEETKAED EKKPKTKKVK **ETTYEWELLN DVKAIWLR**NP KEVTEEEYTK
351 FYHSLAK**DFS DEKPLAWSHF NAEGDVEFKA VLFVPPK**APH DLYESYYNSK
401 KSNLKLYVR**R VFISDEFDEL LPKYLSFLMG LVDSDTLPLN VSR**EMLQQHS
451 SLKTIKKKLI RKALDMIRKI ADEDPDESND KDKK**DVEESG ADNEKK**GQYA
501 K**FWNEFGK**SV KLGIIEGQTN RNRLAKLLRF ETSKSDGK**LT SLDQYISR**MK
551 AGQKDIFYIT GASKEQLEK**S PFLER**LTKKN YEVIFFTDPV DEYLMQYLMD
601 YEDWKGALAS DNVDDVKISN RLADTPCVVV TSK**YGWSANM ER**IMQSQTLS
651 DASKQAYMRG KRVLEINPRH PIIKELRERV VKDPEDESVK **QTAQLMYQTA**
701 **LLESGFLLND PK**DFASRIYS SVK**SSLNVSP DAAVEEEEDI EEPEAEADDN**
751 **EAAAK**DDSDA KDEL

2) luminal-binding protein (XP_016484998.1)

Match to: **NbD049851.1** Score: **135** Expect: **4.8e-009**
NbD049851.1 luminal-binding protein (XP_016484998.1)

Nominal mass (M_r): **73318**; Calculated pI value: **5.14**
NCBI BLAST search of NbD049851.1 against nr
Unformatted sequence string for pasting into other applications

Variable modifications: Carbamidomethyl (C),Oxidation (M)
Cleavage by Trypsin: cuts C-term side of KR unless next residue is P
Number of mass values searched: **109**
Number of mass values matched: **24**
Sequence Coverage: **44%**

Matched peptides shown in **Bold Red**

1 MAGNLRRGSS LVVLAIVLFG CLSALSIAKE EATK**LGTVIG IDLGTTYSCV**
51 **GVYK**NGHVEI IANDQGNR**IT PSWVGFTDSE R**LIGEAAK**NQ AAVNPERTIF**
101 **DVK**RLIGRKF EDKEVQRDMK LVPYKIVNKD SKPYIQVKIK DGETK**VFSPE**
151 **EISAMILTK**M KETAEAFLGK K**IKDAVVTVP AYFNDAQR**QA TKDAGVIAGL
201 NVAR**IINEPT AAAIAYGLDK** KGGEKNILVF DLGGGTFDVS ILTIDNGVFE
251 VLSTNGDTHL GGEDFDQR**IM EYFIK**LIKKK HGKDISKDNR ALGKLRREAE
301 RAKRALSSQH QVR**VEIESLF DGTDFSEPLT RARFEELNND LFR**KTMGPVK
351 K**AMEDAGLEK NQIDEIVLVG GSTR**IPKVQQ LLKDYFDGKE PNK**GVNPDEA**
401 **VAYGAAVQGG ILSGEGGDET KDILLLDVAP LTLGIETVGG VMTK**LIPRNT
451 VIPTKKSQVF TTYQDQQTTV SIQVFEGERS LTKDCRLLGK **FDLTGIAPAP**
501 **RGTPQIEVTF EVDANGILNV K**AEDKGTGKS EKITITNDKG R**LSQEEIER**M
551 VR**EAEEFAEE DKK**VKERIDA RNSLETYVYN MKNQINDKDK LADKLESVEK
601 EKIETATK**EA LEWLDDNQSA EKEDYEEKLK EVEAVCNPII TAVYQR**SGGA
651 PGGGSSEEEE DGHDEL

3) ribulose-1,5-bisphosphate carboxylase/oxygenase large subunit NP_054507.1

Match to: **NbE05064989.1** Score: **229** Expect: **1.9e-018**
NbE05064989.1 ribulose-1,5-bisphosphate carboxylase/oxygenase large subunit (plastid) (NP_054507.1)

Nominal mass (M_r): **52832**; Calculated pI value: **6.55**
NCBI BLAST search of NbE05064989.1 against nr
Unformatted sequence string for pasting into other applications

Variable modifications: Carbamidomethyl (C),Oxidation (M)
Cleavage by Trypsin: cuts C-term side of KR unless next residue is P
Number of mass values searched: **52**
Number of mass values matched: **26**
Sequence Coverage: **50%**

Matched peptides shown in **Bold Red**

1 MSPQTETKAS VGFKAGVKEY K**LTYYTPEYQ** **TKDTDILAAF** **RVTPQPGVPP**
51 **EEAGAAVAAE** **SSTGTWTTVW** **TDGLTSLDR**Y KGRCYRIERV VGEKDQYIAY
101 VAYPLDLFEE GSVTNMFTSI VGNVFGFKAL RALRLEDLRI PPAYVK**TFQG**
151 **PPHGIQVER**D KLNK**YGRPLL** **GCTIKPK**LGL SAKNYGR**AVY** **ECLRGGLDFT**
201 **KDDENVNSQP** **FMR**WRDR**FLF** **CAEALFK**AQS ETGEIK**GHYL** **NATAGTCEEM**
251 **IKR**AVFAR**EL** **GVPIVMHDYL** **TGGFTANTSL** **AHYCRDNGLL** **LHIHR**AMHAV
301 IDRQK**NHGIH** **FR**VLAKALRM SGGDHIHSGT VVGK**LEGERD** **ITLGFVDLLR**
351 **DDFVEQDR**SR GIYFTQDWVS LPGVLPVASG GIHVWHMPAL TEIFGDDSVL
401 QFGGGTLGHP WGNAPGAVAN RVALEACVKA RNEGR**DLAQE** **GNQIIR**EAAK
451 **WSPELAAACE** **VWKEIVFNFA** **AVDVLDK**

4) calreticulin-3-like (XP_016452363.1)

Match to: **NCBI** Score: **87** Expect: **0.00028**
NCBI calreticulin-3-like (XP_016452363.1)

Nominal mass (M_r): **50011**; Calculated pI value: **5.78**
NCBI BLAST search of NCBI against nr
Unformatted sequence string for pasting into other applications

Variable modifications: Carbamidomethyl (C),Oxidation (M)
Cleavage by Trypsin: cuts C-term side of KR unless next residue is P
Number of mass values searched: **87**
Number of mass values matched: **14**
Sequence Coverage: **43%**

Matched peptides shown in **Bold Red**

1 MALSEHKPSR LIFSLETFLL VLLFFTLVSS SASEIFFEES FDDGWRSRWV
51 KSDWKISEGK AGSFK**HTAGT** **WAGDPDDK**GI QTTNDAKHFA VSAK**IPEFSN**
101 **K**NRTLVVQYS IK**FEQDIECG** **GGYIK**LLSGY VNQKKFGGDT PYSFMFGPDI
151 CGTQTKK**LHV** **ILSYQGQNYP** **IKK**ELECETD K**LTHFYTFIL** **RPDASYSIWI**
201 **DGR**ER**DSGSM** **YTDWDILPPR** KIKDVNAK**KP** **ADWEDREYIE** **DPDDIKPEGY**
251 **DSIPR**EIPDR KPKKPNNWDD EEDGMWKPPK VPNPAYRGPW KRKKIKNPNY
301 KGKWK**IPWID** **NPEFEDDPDL** **YVLKPIKYVG** **IEVWQVKAGS** **VFDNILVCDE**
351 **PDYAKQVIQE** **VFANREAEKE** **AFEEAEK**VRK AKEEEEAQRA REEGERRKRE
401 RGRDRHRDRY KKRYHHDYMD DYHDEL

5) Saposin B type domain containing protein (XP_019250726.1)

Match to: **NbD045264.1** Score: **101** Expect: **1.2e-005**
NbD045264.1 uncharacterized protein LOC109229657 (XP_019250726.1)

Nominal mass (M_r): **34402**; Calculated pI value: **6.19**
NCBI BLAST search of NbD045264.1 against nr
Unformatted sequence string for pasting into other applications

Variable modifications: Carbamidomethyl (C),Oxidation (M)
Cleavage by Trypsin: cuts C-term side of KR unless next residue is P
Number of mass values searched: **54**
Number of mass values matched: **14**
Sequence Coverage: **46%**

Matched peptides shown in **Bold Red**

1 MAKVELLLLL SLMVITTWFP ISHCAKKPVG VARKEDIPYI KCQVCEK**LAY**
51 **QLYNGVQNK**R AEISPKK**ISE** **YQIIEISENV** **CNLKKQEADW** **ILKIDIVEQG**
101 **DRLELVEQDS** **EGQCNSECK**T IERACQDVMD YSDTDVAEYL YKHNPDLDSL
151 KSFLCKDLTK ACSKASPPVP K**NRAPGEPFV** **SK**SSKEAEME KLMR**SMQDMP**
201 **GAPGMQMYSR** **EDLMNQNFGD** **EDADGDDDDD** **DEGGFPSK**LG KVLKGKERKK
251 NDWKQWIAK**G** **IQDTSEVLK**T HAYRVSHRMR **NWWGAR**K**VQW** **KK**NSKTSKGE
301 L

Figure S3. Loading controls to CRT3 – *Nt*Phyt *in vitro* binding experiments.

(a). Lysates of His-CRT3-overproducing *E. coli* cells (CRT3) and of vector-only transformed *E. coli* cells (vector) were loaded onto 200 μL Ni-NTA agarose resin. After washing, resin-bound proteins were eluted with a buffer containing 100 mM EDTA and resolved by SDS electrophoresis in a 10% polyacrylamide gel. Proteins were visualized after Coomassie Blue staining. Image of a heavily overloaded gel is shown to demonstrate equal amounts of bacterial proteins non-specifically bound to CRT3-containing and control resins. M, molecular weight protein markers. Arrow indicates position of His-CRT3.

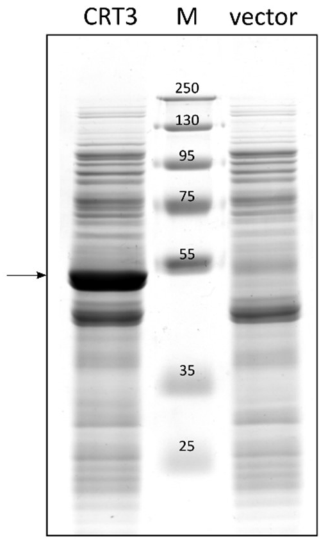

(b). Four equal aliquots 1/5 of CRT3-containing and control resins described in **(a)** were taken. Three of them were incubated with *Nt*Phyt to assess the interaction. From the 4th aliquots, the resin-bound proteins were eluted with a buffer containing 100 mM EDTA and analyzed by SDS-gel electrophoresis with Coomassie Blue staining to evaluate the amount of His-CRT3 in the samples. M, molecular weight protein markers (approx. 2 μg protein per band). Arrow indicates position of His-CRT3. Also, a band of non-specifically bound ~40-45 kDa bacterial protein serves as a loading control.

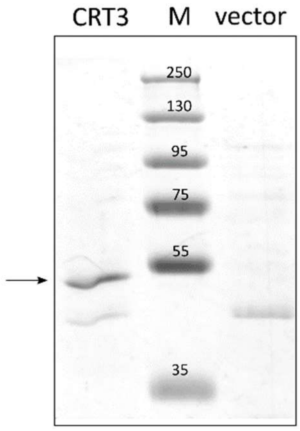

Table S1. List of the primers used in this study.

#	Primer name	Sequence
1	TurboID_Bam_dir	GTGGATCCTCTGGGAGGTTCTAAAGACAATACTGTGCCTCTG
2	TurboID_His6_z_Sac_rev	GGGAGCTCTTAATGGTGATGATGATGGTGCCGCGGCTTTTCGGCAGACCGCAG
3	CRT_Kpn_Nco_dir	CAAGGTACCATGGCTCTCTCTGAGCATAAAC
4	CRT_Bam_rev	CAAGGATCCTTAAAGTTCATCATGATAGTCATC
5	CRT_LF_Apa_Nde_dir	GAGGGCCCGCATCTCATATGTCTGAGATTTTCTTTGAAGAAAG
